# A review of characteristics and outcomes of Australia’s undergraduate medical education rural immersion programs

**DOI:** 10.1186/s12960-018-0271-2

**Published:** 2018-01-31

**Authors:** Belinda G. O’Sullivan, Matthew R. McGrail, Deborah Russell, Helen Chambers, Laura Major

**Affiliations:** 1 Monash University School of Rural Health, Office of Research, Level 3, 26 Mercy St, PO Box 666, Bendigo, VIC 3550 Australia; 2 Monash University School of Rural Health, Northways Road, Churchill, VIC 3842 Australia; 3 Monash University School of Rural Health, 3 Ollerton Ave, Newborough, VIC 3825 Australia; 4 Monash University School of Rural Health, Clayton, Australia

**Keywords:** Rural immersion, Doctors, Rural practice, Rural supply, University, Medical school, Rural program

## Abstract

**Background:**

A key strategy for increasing the supply of rural doctors is rurally located medical education. In 2000, Australia introduced a national policy to increase rural immersion for undergraduate medical students. This study aims to describe the characteristics and outcomes of the rural immersion programs that were implemented in Australian medical schools.

**Methods:**

Information about 19 immersion programs was sourced in 2016 via the grey and published literature. A scoping review of the published peer-reviewed studies via *Ovid MEDLINE* and *Informit* (2000–2016) and direct journal searching included studies that focused on outcomes of undergraduate rural immersion in Australian medical schools from 2000 to 2016.

**Results:**

Programs varied widely by selection criteria and program design, offering between 1- and 6-year immersion. Based on 26 studies from 10 medical schools, rural immersion was positively associated with rural practice in the first postgraduate year (internship) and early career (first 10 years post-qualifying). Having a rural background increased the effects of rural immersion. Evidence suggested that longer duration of immersion also increases the uptake of rural work, including by metropolitan-background students, though overall there was limited evidence about the influence of different program designs.

Most evidence was based on relatively weak, predominantly cross-sectional research designs and single-institution studies. Many had flaws including small sample sizes, studying internship outcomes only, inadequately controlling for confounding variables, not using metropolitan-trained controls and providing limited justification as to the postgraduate stage at which rural practice outcomes were measured.

**Conclusions:**

Australia’s immersion programs are moderately associated with an increased rural supply of early career doctors although metropolitan-trained students contribute equal numbers to overall rural workforce capacity. More research is needed about the influence of student interest in rural practice and the duration and setting of immersion on rural work uptake and working more remotely. Research needs to be more nationally balanced and scaled-up to inform national policy development. Critically, the quality of research could be strengthened through longer-term follow-up studies, adjusting for known confounders, accounting for postgraduate stages and using appropriate controls to test the relative effects of student characteristics and program designs.

**Electronic supplementary material:**

The online version of this article (10.1186/s12960-018-0271-2) contains supplementary material, which is available to authorized users.

## Background

Universal access to medical services is integral to the achievement of the United Nations’ sustainable development goals [1]. One strategy to improve the supply of rural doctors is providing rural immersion experiences for undergraduate medical students. This has underpinned a growth of rural immersion programs for medical students globally since the 1970s [2–7].

Australia was the first country to implement a national policy offering more and longer rural immersion opportunities to undergraduate medical students through its Rural Clinical Schools (RCS) program, which began in 2000. This policy is one of many strategies aiming to increase the supply of rural doctors in Australia [8, 9]. The policy funds medical schools to (1) select students already familiar with rural areas by requiring a minimum 25% of the enrolled cohort to have a childhood rural background and (2) provide opportunities for more students to become attuned and interested in rural practice by requiring at least 25% of enrolled students do at least 1 year of clinical learning in rural locations [10]. By 2009, 19 medical schools had implemented (ongoing) rural immersion programs (called “immersion programs” hereafter).

A wide range of immersion programs were implemented to suit different medical school structures, jurisdictions and regional contexts whilst also meeting government funding requirements. As yet, there is no consolidated information about the characteristics and outcomes of these programs. A range of universities have researched the outcomes of their own immersion programs; however, there is a notable gap in synthesis of the range, quality and strength of the evidence that has emerged at a national level. This has important implications for informing policy and program development.

Previous literature reviews about rural immersion have been based on narrative approaches, limited to relatively small North American programs or programs, mainly focused on overall impact, with less attention to issues of student selection, program design and outcomes by doctors’ postgraduate stages [11–13]. Having 19 immersion programs in one country provides a unique chance to explore immersion program evidence in more detail, within a consistent health system context.

This study aims to describe the characteristics and outcomes of rural immersion programs in Australian medical schools, including the different effects based on student characteristics or the characteristics of immersion.

## Methods

Information about Australia’s 19 universities offering immersion programs during undergraduate medical training was extracted from peer-reviewed and grey literature including university websites. Rural immersion was defined as clinical training occurring for at least one academic year in non-metropolitan locations (levels 2 to 5 on the Australian Standard Geographical Classification-Remoteness Areas (ASGC-RA) schema). Rural locations included both regional centres and small rural and remote towns. Data were systematically collected about program goals, methods of student selection, access to rural immersion training streams, duration of rural immersion and whether or not immersion was in general practices in rural towns.

A scoping review was selected as an appropriate method to map, collate and summarise the literature. As opposed to systematic reviews which are best suited for narrow research questions and particular types of evidence like randomised controlled trials, scoping reviews are better suited for exploring broad topics like rural immersion and can include a broader range of study types [14]. The five stage framework by Arskey and O’Malley was applied, starting with a broad question and key terms to enable breadth of coverage of available local research [14].

Peer-reviewed published literature were accessed via a comprehensive keyword search of *Ovid MEDLINE* and *Informit* from 2000 to 2016. Terms included were (Clin* School* OR Med* School*) AND (rur* placement OR rur* training OR rur* immersion OR rur* exposure OR rur* clinical placement) AND (Internship OR Medic* OR doctor* OR graduate).

To capture the full range of available information on local immersion programs, additional subject searches were undertaken within selected Australian health journals (Medical Journal of Australia, Rural and Remote Health and the Australian Journal of Rural Health) using searches for the terms: *university*, *rural clinical school*, *rural training*, *graduates.* Similar subject searches were conducted within international journals (BMC Medical Education and Medical Education) using the term *rural training*. The reference lists of retrieved articles and published literature reviews about rural clinical training were reviewed for additional relevant articles, and articles on the topic already known to the researchers were also eligible for inclusion.

Titles and abstracts were appraised by two researchers. Studies were included if they related to outcomes of rural immersion during undergraduate medical training in Australia 2000–2016 or reported rural practice or intention for rural practice (for individual medical schools that had limited or no research about rural practice outcome). Articles were excluded if they were qualitative, of non-medical professionals, related to postgraduate training, or were from outside Australia. All included articles were read by three authors in full text.

Applying the scoping review method, data were charted and collated through regular face-to-face meetings, to summarise major findings, discussion points and limitations. To extend on the scope of previous reviews (mainly related to the impact of immersion), this review specifically explored outcomes at different postgraduate periods (internship, as the first year of supervised hospital-based practice, or other early career stages during the first 10 postgraduate years), and the characteristics of the students and the types of immersion. Each study was reviewed by location, population, objectives, methods, outcomes and strengths and limitations. The evidence was then charted in two categories for informing policy development and program decisions: (1) the overall effects of immersion on rural work outcomes and (2) whether effects varied by student characteristics. For both categories, the duration of immersion was considered.

### Study selection

**Fig. 1:**
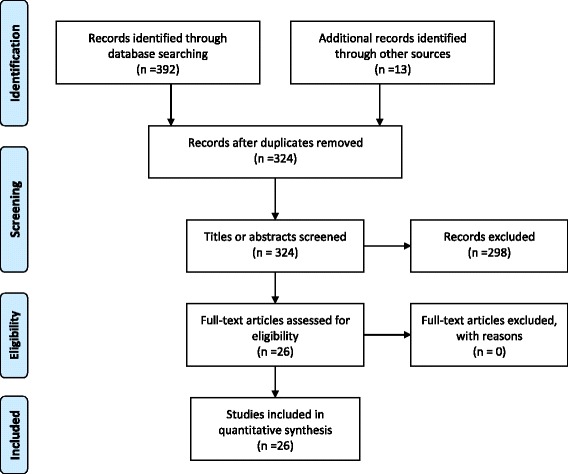
PRISMA 2009 flow diagram

Overall 392 studies were identified: 45 were identified from *Ovid MEDLINE* and 347 from *Informit*. An additional 12 were from direct searching and one that was known to authors. After screening, 26 eligible studies were included (Fig. 1).

## Results

The characteristics of the immersion programs varied widely (Table 1). Four stated a clear program mission to train rural doctors. Two such schools actively enrolled more than the required quota of 25% rural-background students. Three also linked rural-background students with rural immersion opportunities and used active processes like interviews and written applications to screen students for their interest in rural practice.

**Table 1:** Summary of program characteristics by university medical school

Medical school	Proportion enrolled students with rural-background^a^	Target rural immersion opportunities for rural-background students	Actively screen for interest in rural practice^b^	Immersion duration	Rotations into general practice in small communities
Stated a mission to train rural doctors					
Deakin	25%	No	Placement preferences only	1 year	Yes
Flinders	25%	Yes	Yes	1 year	Yes
James Cook	40%	Yes	Yes	Wholly rural^c^	Yes
Wollongong	Up to 70%	Yes	Yes	At least 1 and up to 3 years	Yes
Did not state a mission to train rural doctors					
Adelaide	NS	NS	NS	1 year	Yes
ANU	NS	NS	Placement preferences only	1 year	NS
Bond	NS	NS	NS	1 year	NS
Griffith	25%	No	Yes	At least 1 and up to 3 years	Yes
Melbourne	25%	No	Yes	At least 1 and up to 3 years	Yes
Monash	25%	No	Placement preferences only	At least 1 and up to 3 years	Yes
Newcastle	NS	NS	NS	At least 1 and up to 3 years	Yes
Notre Dame	NS	NS	Yes	1 year	NS
Queensland	NS	NS	Placement preferences only	At least 1 and up to 3 years	No
Sydney	NS	NS	Placement preferences only	1 year	NS
Western Sydney	NS	NS	NS	1 year	Yes
University of New England	NS	NS	NS	At least 1 and up to 3 years	Yes
Tasmania	30%	No	Placement preferences only	Wholly rural^c^^d^	Yes
UNSW	25%	No	Placement preferences only	At least 1 and up to 3 years	NS
Western Australia	25%	NS	Yes	1 year	Yes

*NS* not stated, *Placement preferences* students nominate their preference for a rural placement annually. At Monash Universitylimited students can also self-nominate for rural training at course entry.

^a^The quota required by the Australian government policy is 25% in 2016

^b^*Yes*—interest screened at course entry or during the course, prior to the immersion opportunity

^c^Students undertake whole medical course in rural areas (mostly in large coastal regional centres)

^d^Students undertaken whole medical course as per ^c^. with provision for 1 year in a town of 25,000 population

Of the 15 other schools that did not specify a mission for training rural doctors, only one enrolled more than 25% rural-background students; no schools aimed for such students to be streamed into rural immersion opportunities. Four used interviews to screen student interests in rural practice in order to allocate a sub-set of students to rural immersion streams. Another six used student placement preferences to allocate rural immersion streams.

A range of durations of immersion were identified. Nine schools offered a maximum immersion of 1 year, eight up to 3 years, and two offered a wholly rurally based course, mainly based in large coastal regional centres. Thirteen schools offered more than 6 months of immersion in small rural towns, attached to general practices in the community (Table 1).

The 26 studies included in the scoping review are described in Additional file 1: Appendix 1.

The studies originated from 10 of the 19 medical schools and 16/26 were from two states (12 studies from Queensland and 4 from Western Australia). Only three studies were from the most populated states of New South Wales and Victoria. Two medical school studies and one national study reported the outcome as intention for rural practice at graduation rather than actual practice.

Table 2 summarises the key findings of these studies in relation to the overall effect of immersion on rural work outcomes.

**Table 2:** Rural practice at internship and later postgraduate stages by duration of rural immersion during undergraduate medical training

Duration of immersion	Rural internship (PGY1)^a^	Rural practice at mixed or later postgraduate stages^a^
1 year	21% (*n* = 20/94) rurally immersed versus 4% (*n* = 15/342) (metropolitan-trained) [16]	
	18% (*n* = 13/72) rurally immersed (no control group) [23]	28% (*n =* 16/58) rurally immersed in rural practice in PGY2 (no control group) [23]
	17% (*n* = 63/367) rurally immersed (no control group) [24]	16% (*n* = 42/258) rurally immersed versus 5% (*n =* 36/759) wholly metropolitan-trained worked rurally when surveyed (spanning PGY3–10) ^(#)^ (multivariate analysis, adjusted for rural background, sex, age) [26]
		79% (*n =* 48/61) rurally immersed working in outer regional/remote versus inner regional locations in PGY3–10, versus 46% (*n =* 91/200) historical controls and 52% (*n =* 33/63) metropolitan-trained ^(#)^ (multivariate analysis, adjusted for rural background, sex) [27]
1 year (town of population 25,000 versus whole course in large regional centre)		57% (*n =* 106/185) rurally immersed versus 49% (*n* = 333/684) regional-trained worked rurally when surveyed (spanning PGY1–11) ^(#)^ (univariate analysis only) [28]
Up to 2 years		42% (*n =* 115/276) rurally immersed versus 19% (*n =* 90/478) wholly metropolitan trained when surveyed (spanning PGY1–10) ^(#)^ (multivariate analysis, adjusted for most key confounders) [29]
Wholly rural	67% (*n =* 194/292) rurally immersed compared with 32% (*n =* 222/720) from medical schools in same state and 17% (*n =* 374/2174) from other Australian medical schools ^(#)^ (univariate level only) [15]	54% (*n* = 95/175) rurally immersed working in rural location by PGY3 (no control group) [25]

See Additional file 1: Appendix 1 for description of studies and their limitations. PGY1 is the internship year in Australia, when doctors have provisional registration and work under supervision to gain their general registration by PGY2

*PGY* postgraduate year following the completion of the medical course

^a^(#)Statistically significantly associated, see Additional file 1: Appendix 1 for *P* values and respective odds ratios and confidence intervals

A high proportion of students who trained wholly in rural areas (67%, *n* = 194/292) worked as rural interns [15], although a higher proportion of graduates of other universities in the same state (offering 1–2-year immersion) also undertook rural internships (32%) than an average from other states (17%) (Table 2). Another program found that 21% (*n* = 20/94) of students immersed for 1 year were working as rural interns compared with 4% (*n* = 15/342) of metropolitan-trained controls, though the study did not adjust for potential confounders [16]. A range of studies reported intern work outcomes or rural intent of graduates but had small numbers and lacked control groups [17–22].

Several studies showed that rural work varies by early career stages, but the influence of immersion programs on this variation was poorly differentiated due to a lack of control groups [23, 24]. An average proportion of 22% of early career graduates who had been immersed for 1 year worked in rural areas in one small study of 72 rural clinical school graduates, the highest proportion in PGY2, 28% (*n* = 16/58) [23]. Another medical school program suggested that 17% (*n* = 63/367) of early career graduates worked in full-time rural practice, although a further 22% (*n* = 81/367) had worked rurally at some point in the preceding 10-year period, (56%, *n* = 45/81 for only 2–12 weeks) [24]. Another wholly rurally trained cohort showed a gradual increase in the proportion of graduates working in the city comparing the cohort at internship (34%, *n* = 59/175) to the third to seventh postgraduate year (46%, *n* = 80/175). Location at PGY 5–7 had a univariate association with type of postgraduate specialist training being undertaken [25].

Only four studies explored rural practice outcomes beyond internship that included metropolitan-trained control groups (Table 2). From one school, 16% (*n* = 42/258) of students immersed for 1 year worked rurally in their early career compared with 5% (*n* = 36/759) of those who trained in metropolitan areas; however, no adjustment was made for potential confounders like rural return of service obligations [26]. Rurally immersed students within this medical school were additionally more likely to work in more geographically remote locations: 79% (*n* = 48/61) in outer regional or remote areas compared with 46% (*n* = 91/200) of historical controls and 52% (*n* = 33/63) of students who were wholly metropolitan trained. Nevertheless, more doctors working in outer regional (*n* = 86) or remote (*n* = 41) locations were from the metropolitan-trained group rather than the immersed group (*n* = 31 and *n* = 17 respectively) [27]. Another study showed that 57% (*n* = 106/185) of students immersed for 1 year in a smaller town worked rurally compared with 49% (*n* = 333/684) trained wholly in major regional centres. This study also did not account for any potential confounders of rural practice [28]. Finally, a large cross-sectional study, which did adjust for a range of key confounders, found 42% (*n* = 115/276) of students immersed for 1 to 2 years worked rurally in their early careers compared to 19% (*n* = 90/478) trained in metropolitan areas. However, there was the potential for bias from using a self-reported rural outcome measure, via survey with a 48% response rate [29].

Table 3 summarises the key findings related to students’ rural backgrounds and their interest in rural practice.

**Table 3:** Rural practice at internship and later postgraduate stages based on immersing students with different characteristics during undergraduate medical training

Duration of immersion	Rural internship (PGY1)	Rural practice at mixed or later postgraduate stages^a^
1 year	67% (*n =* 31/46) those immersed *based on interest* (written application and interview) versus 15% (*n =* 63/426) metropolitan-trained (either did not express interest/ were not selected) [30]	21% (*n* = 13/63) of those with a *rural background* were practising rurally in PGY3–10 versus 15% (*n* = 29/195) of metropolitan-background students (immersing rural and metropolitan-background students both significantly associated with working rurally ^(#)^ in multivariate modelling, adjusted for rural background, sex, and age) [26]
	10% (*n* = 7/71) *rural-background* students versus 7% metropolitan-background (*n* = 24/331) [16]	
Up to 2 years		61% (*n* = 73/119) *rural background* versus 27% (*n* = 42/157) practised rurally in PGY1–9 when both groups had been rurally immersed, 1- or 2-year immersion significantly associated with rural-background students working rurally, not metropolitan background, though 2 years immersion for metropolitan background approached significance ^(#)^ in multivariate modelling, adjusted for most key covariates [29]
		22% (46/176) of *non-conscripted immersed students*, versus 4% (*n* = 8/31) non conscripted working rurally in PGY2–9 (approximate as date of survey not given), ^(#)^ univariate analysis only [31]
Wholly rural	69% (*n* = 284/413) *rural background* versus 43% (*n =* 3/825) metropolitan background [33]	

See Additional file 1: Appendix 1 for description of studies and their limitations. PGY1 is the internship year in Australia, when doctors have provisional registration and work under supervision to gain their general registration by PGY2

*PGY* postgraduate year following completion of the medical course

^a^(#)Statistically significantly associated, see Additional file 1: Appendix 1 for *P* values and respective odds ratios and confidence intervals

One medical school selecting a small number of students for immersion based on their rural practice interests (by written application and interview) found that 67% (*n* = 31/46) undertook rural internships, compared with 15% (*n* = 63/426) who were wholly metropolitan-trained (Table 3). Multinomial logistic regression analysis (adjusting for rural background, gender and cohort, but not rural return for service obligations) suggested that rural immersion of interested students, and not rural background, was significantly associated with rural internship, although the immersion sub-groups were very small [30].

Only three studies explored whether immersion by choice improved rural practice outcomes (Table 3). One was a small cross-sectional study, which was limited to reporting intent for rural practice as the outcome [20]. The second, a univariate analysis of small sub-groups only, found that 22% (*n* = 46/176) of students not conscripted to participate in rural immersion were working in rural areas compared to 4% (*n* = 8/31) who were conscripted [31]. The final study was also limited to measuring rural practice intent as the outcome but was a large national cohort study using multiple logistic regression modelling (adjusting for key covariates except rural return of service obligations and international students required to work in rural areas after graduation). It found that a course commencement self-reported preference to work in a rural area as a doctor was associated with increased intention to practice rurally at course completion (adjusted OR 6.0, 95% CI 4.8–7.6). The study identified other independent predictors of rural practice at course completion, including rural background and later (after third year) remote immersion (adjusted OR 2.3, 95% CI 1.6–3.4; and 1.8, 95% CI 1.3–2.5, respectively), though total immersion duration was difficult to ascertain [32].

Only four studies explored immersion outcomes stratifying by childhood rural background (Table 3). Of these, two reported internship outcomes only. One showed that 69% of rural-background graduates undertook rural internships (*n* = 284/413) compared with a small metropolitan-background group (43%, *n* = 35/82) when both groups were wholly rurally trained, though it was based on univariate analysis only [33]. A study of another program showed similar rural internship outcomes after 1-year immersion for rural- (10%, *n* = 7/71) and metropolitan-background students (7%, *n* = 24/331), not significantly different (*P* = 0.455), whereas rural immersion was associated with rural internship, but this was based on univariate analysis only (21%, *n* = 20/94 immersed versus 4%, *n* = 15/342 not immersed *P* < 0.001) [16].

Two other larger studies reported outcomes for graduates beyond internship, with differing results. The first reported that following 1- to 2-year immersion, 61% (*n* = 73/119) of rural-background students practised rurally compared with 27% (*n* = 42/157) of metropolitan-background students [29]. The study adjusted for a range of key covariates except rural return for service obligations and international student status. This study also found that 1-year and 2-year immersion were independently associated with increased odds of rural-background students practising rurally (OR 4.4, 95% CI 2.4–8.3 and OR 7.1, 95% CI 3.6–14.1 respectively) in multivariate analysis compared to a metropolitan-background and metropolitan-trained control group [29]. Two-year immersion for metropolitan-background students also had a stronger association with rural practice than 1 year but this was not statistically significant.

The second of the larger studies showed that 1 year of immersion was associated with increased odds of rural practice for both rural- (OR 7.5, 95% CI 3.5–15.8) and metropolitan-background students (OR 5.1, 95% CI 2.9–9.1) compared with metropolitan-trained controls [26], with an independent effect of rural background (OR 4.2, 95% CI 1.8–9.2); however, it also did not adjust for rural return of service obligations [26].

An additional study, with no metropolitan-trained control group and based on univariate anlayses only, suggested that being raised in a more geographically remote community doubled the proportion of wholly rurally trained students returning to such locations at internship and working there in early career, compared with students without this upbringing [34].

Four studies explored the influence of duration, timing or type of rural immersion on subsequent rural practice outcomes, two of which only followed students to internship. Greater proportions of students who were immersed for more than 20 weeks undertook rural internships, though no adjustment for key confounders was done [35]. In another study, 18 months of immersion was associated with 100% of students (*n* = 6) taking up a rural internship compared to 81% (*n* = 21/26) of students immersed only for 1 year (not statistically tested and small sample size) [30]. Two other cross-sectional studies explored outcomes beyond internship. The first found the duration of rural immersion increasing from 1 to 2 to 3 years increased rural work in early career but the sub-groups were too small for reliable statistical analysis [31]. The second, a larger cross-sectional study, showed stronger odds of rural practice with increased duration of immersion (from 1 to 2 years) (OR 1.84, 95% CI 1.21–2.82 and OR 2.71, 95% CI 1.65–4.45 respectively), based on adjusted multiple logistic regression analysis [29].

Only two studies explored the effects of the setting and remoteness of rural immersion on subsequent rural practice. One found the odds of rural work/training by the fourth postgraduate year increased 19-fold for students immersed in small rural general practices for 1 year and fourfold if immersed in a large regional referral hospital relative to graduates who wholly trained in metropolitan areas, although the immersion sub-groups were small and the rural work outcome was imprecise [36]. The other study, based on a wholly rural program, found an association between the location of immersion and internship and current work locations [37].

### Quality

An assessment of the strengths and limitations of individual papers is described in Additional file 1: Appendix 1.

The overall quality of the 26 studies was low. Many only used univariate analyses and thus inadequately controlled for confounding variables. Although more recent studies were based on larger populations of graduates or larger sample sizes, with longer follow-up and better adjustment for confounders, none sufficiently accounted for all of the main measureable potential confounders, particularly students with rural return of service obligations or international students who are more likely to work rurally due to a policy that places limitations on where they can work to access a provider (billing) number. Studies also provided no justification for the postgraduate period in which rural practice outcomes were measured. Some cross-sectional designs relied wholly on self-reported rural practice and had relatively low response rates. Most studies did not explore distribution within rural geographies, instead analysed rural work outcomes at a binary level (rural or metropolitan). Finally, many studies did not have sufficient control groups of non-immersed students or cohorts undertaking different types of immersion, to test program effects.

## Discussion

The Australian government has made a significant investment in supporting rurally located undergraduate medical education, since 2000. A wide variety of minimum 1-year immersion programs are occurring as a result. This review indicates that rural immersion is consistently associated with an increased proportion of Australian medical graduates practising rurally during their early careers, irrespective of how the immersion is structured. However, the quality of evidence is generally low; there needs to be more multivariate modelling to account for key potential covariates, and better use of control groups, so that the effects of immersion programs can be better isolated.

The review findings suggest that students with rural backgrounds are more likely to pursue rural practice with immersion opportunities. Further, students undertaking longer duration immersion, including those from metropolitan areas, may be more likely to work rurally. However, the evidence about program designs that are most effective remains relatively under-developed. The broad range of immersion program designs evident across Australia provides a unique opportunity to extend the research about these effects. Yet all studies except one (a national study that only used rural practice intent as the outcome) have been single-institution studies (usually with a single type of immersion program). This has restricted the assessment of design phenomena. Scaling up the research is likely to reduce institutional research bias and promote better sub-group comparisons. Specific areas where more evidence is needed include the outcomes of immersing students who are interested in rural practice, providing a variety of lengths and timings of immersion in different geographical remoteness and clinical settings. Further, the review identified that about half of rural doctors are wholly trained in metropolitan areas. The reasons why this group pursues rural practice in equal numbers bears further analysis [38].

With respect to informing national policy, the evidence could be more geographically balanced, rather than emanating mainly from two states. A major contributor of the research included in this review was a single medical school that manages a wholly rural medical program but lacks a metropolitan-trained control group to test the influence of immersion independently of other program and student cohort characteristics. The internship outcomes of this program compared with aggregated outcomes of (1) medical graduates in the same state and (2) other Australian medical schools, suggest that there may be jurisdictional differences that relate to rural practice outcomes. These may relate to more positive outcomes in states with higher proportional rural population, larger coastal regional centres, and more state-based rural return of service schemes and scholarships.

As an important quality issue, future studies in this area should better justify the time-point at which rural practice outcomes are measured. This is pertinent given several included studies identified the high mobility of early career doctors. Many studies measured rural practice at internship. However, Australian internships are allocated through an increasingly competitive state-based matching process, based on hospital and student preferences, not wholly reflecting graduate choice of work location and predominantly limited to hospitals with positions. Beyond internship, in Australia, specialty training generally occurs from the third postgraduate year with few non-general practice (GP) specialty training places available in rural areas. This requires most non-GP specialty registrars to live and work in metropolitan areas during their early careers. Conversely, in GP specialty training, rural training opportunities are over-represented. The main period when doctors may independently choose rural practice is after specialising, typically around their sixth to eighth postgraduate year. This demands longer-term tracking, including consideration of postgraduate specialty choices.

Limiting the scope of the review to Australia has restricted the ability to generalise the findings beyond Australia. However, important insights about Australia’s unique national rural immersion policy can inform international directions for research, policy development and program decisions. Many countries have similar workforce training systems, geographical contexts and significant population health issues that might be comparable to those found in Australia. Comprehensive search strategies and expert database advice was used to identify the material supporting this review; however, the broad indexing of this topic may have limited the articles able to be identified by routine database searches. It is possible that this study missed relevant material, although this was counteracted by including studies known to the authors via their rural health workforce research networks.

## Conclusion

Australia’s rural immersion policy has resulted in a wide range of structured programs in medical schools nationally. This review suggests that the policy is tracking well with respect to its intent. Despite the varied structure of different immersion programs, rural immersion is consistently associated with increased rural supply of early career doctors in Australia. Evidence suggests that selecting rural-background students and providing longer immersion for metropolitan-background students may further increase program effectiveness. Students trained in metropolitan areas still remain important for boosting overall rural workforce capacity. The focus of further research needs to be about the influence of student characteristics and the duration and setting of immersion on rural work uptake and working more remotely. Research could be more nationally balanced and scaled-up, include repeated and longer follow-up of graduates at justified postgraduate training stages and apply appropriate multivariate methods and control groups to closely evaluate program selection and design effects.

## Additional file

Additional file 1: Appendix 1: Description of papers included in the review^a^ [39–41]. (DOCX 36 kb)

## Abbreviations

ASGC-RA: Australian Standard Geographical Classification-Remoteness Areas
CI: Confidence interval
GP: General practice
OR: Odds ratio
PGY: Postgraduate year following completing medicine
RCS: Rural Clinical School
