# Supplementary material for: A review of characteristics and outcomes of Australia’s undergraduate medical education rural immersion programs
Source: Hum Resour Health. 2018 Jan 31;16:8. doi: 10.1186/s12960-018-0271-2 (PMC5793366; doi:10.1186/s12960-018-0271-2)
Supplement: Additional file 1: — Appendix 1: Description of papers included in the reviewa [39–41]. (DOCX 36 kb) [file 12960_2018_271_MOESM1_ESM.docx]

**Additional file 1: Appendix 1**: Description of papers included in the review ^a^

| **Author year & place** | **Title** | **Population** | **Objectives & methods** | **Outcomes** ^b^ | **Strengths and Limitations** ^c^ |
| --- | --- | --- | --- | --- | --- |
| Clark et al 2013 (16)  New South Wales | Medical graduates becoming rural doctors: rural background versus extended rural placement | 821 medical students commencing the course at University of Sydney 2005-2007, immersed or not | Self-reported course entry and exit questionnaire and workforce registration data used to identify whether intention for rural practice at course commencement and rural immersion was related to rural internship. Univariate analyses only. | Immersed students significantly more likely to accept a rural internship (21%, n=20/94) versus non-immersed 4%, n=15/342) (P <0.001). Rural background not associated with rural internship versus metropolitan-background (10%, n=7/71 vs 7%, n=24/331, P =0.455). | 55% students responded to both surveys (n=448). No adjustment for key confounders. Did not specify how handled international students. Outcomes for internship only. |
| Eley et al 2006 (17)  Queensland | Does recruitment lead to retention? - Rural Clinical School training experiences and subsequent intern choices | 26 final year medical students from University of Queensland Rural Clinical School (RCS) in 2005, all rurally-immersed | An end of course 63-item questionnaire to explore immersion experience and internship location choice. No statistical testing. | 23%, n=6/25 rurally-immersed students elected to stay in their respective rural teaching hospitals for internship. | Only 26 were immersed in 2005 cohort, n=25 responded. No metropolitan-based control group or adjustment for key confounders. |
| Eley et al 2007 (18)  Queensland | Will Australian rural clinical schools be an effective workforce strategy? Early indications of their positive effect on intern choice and rural career interest | Final year medical students from University of Queensland RCS 2005-2006, all rurally-immersed | Self-reported pre and post immersion questionnaire exploring among other things, uptake of rural internship. No statistical testing. | 45%, n=20/44 students who undertook rural immersion in 2005 or 2006 subsequently chose rural internships. | Limited numbers (n=44). No metropolitan-based control group or adjustment for key confounders. |
| Eley et al 2009 (39)  Queensland | Self-sufficiency in intern supply: the impact of expanded medical schools, medical places and rural clinical schools in Queensland | Interns employed in Queensland 2003-2008 | Health department intern data 2003-2008 and university records to explore proportional supply of Queensland interns (by RRMA (remoteness)) by two Queensland medical schools or other sources and relative to immersion experience. Univariate analyses only. | Two Queensland medical schools supplied 72% and 84% of Queensland interns in 2007 and 2008. Rurally-immersed graduates were more likely to work in hospitals in RRMA 3 versus RRMA 1 in 2007 (8.8 [4.6–16.7]; P<0.0001) and 2008 (6.5 [3.5–12.2]; P<0.0001) relative to those wholly metropolitan-trained. | Only showed number of University of Queensland (UQ) students in internships 2003 to 2008 but total graduating cohort number unknown. Only reported immersion outcomes for two years (2007-2008). Limited to Queensland interns. No adjustment for key confounders. |
| Eley et al 2012 (40)  Queensland | A decade of Australian Rural Clinical School graduates – where are they and why? | 180 medical students from University of Queensland RCS (entering the workforce 2002-2009), all rurally-immersed | A cross-sectional retrospective survey in 2010 (when in PGY 2-9) to explore early career choices related to rural immersion. | 39%, n=38/97 respondents currently working in rural locations. | 54% response rate (n=97/180) to the survey question about current location. No metropolitan-trained control group. No statistical testing. |
| Forster et al 2013 (31)  New South Wales | Additional years of Australian rural clinical school undergraduate training is associated with rural practice | 315 medical students from University of New South Wales RCS 2003-2010, all rurally-immersed | Online cross-sectional retrospective survey (no survey date) to explore whether 1-3 years of RCS training influences current, preferred current and intended locations of graduates. Univariate analyses only. | Overall, 26%, n=54/207 of those rurally-immersed 1-3 years’ currently worked in rural areas: 15%, n=6/40 if immersed for one year, 23%, n=23/100 for two years and 37%, n=25/67 for three years (P=0.024). 4%, n=8/31 who were conscripted currently worked rurally, versus 22% n=46/176 not conscripted, univariate odds (2.5 [1.0-6.3], P=0.015) | 66% (n=207/315) useable responses. Small sub-groups analysed. Potential bias via self-reported outcome. Univariate association of rural background but no adjustment for key confounders. Did not specify how handled international students. No metropolitan-trained control group. |
| Isaac et al 2014 (19)  New South Wales | The influence of rural clinical school experiences on medical students’ levels of interest in rural careers | 175 medical students from University of New South Wales RCS in 2013, having been immersed for 1 to 3 years | Pre and post rural immersion experience self-reported responses to cross-sectional survey exploring factors associated with intent to practise in a rural location among students who spent different periods training in the RCS. Univariate analyses only. | Factors associated with rural intent practice intent were rural background 84%, n=98/118 (χ2 = 28.4, P<0.001), two or more previous years of rural immersion 67%, 79//118 (χ2=9.0, P = 0.003), and preference for a rural internship 54%, n=64/118 (χ2=17.8, P< 0.001). | Response rate 67%, n=118/176. Limited to rural practice intention, not actual rural practice outcomes. No adjustment for key confounders – did multivariate modelling but covariates model and outcomes not stated. International students excluded. No metropolitan-trained control group. |
| Jamar et al 2014 (23)  South Australia | Early career location of University of Adelaide rural cohort medical students | 127 medical students from University of Adelaide RCS 2003-2010, all rurally-immersed | Self-completed online 28-item questionnaire (2012), exploring rural background, rural immersion experience and career choices. Univariate analyses only. | 18%, n=13/72 worked as interns in rural area. 28%, n=16/58 worked PGY2 rural. An average of 22% of respondents worked in a rural area for the postgraduate years studied. More than half (56%, n=40/72) spent 3+ months postgraduate time in rural location. | Response rate 57%, n=72/127 had location information. International students excluded. Reported frequencies only, no statistical testing. No adjustment for key confounders. No metropolitan-trained control group. |
| Jones et al 2014 (32)  National | Are rural placements positively associated with rural intentions in medical graduates? | 4486 students commencing medicine in one of 21 Australian universities 2005 – 2008 | Longitudinal study using university course commencement and completion questionnaires, exploring factors influencing rural work intention at course completion. Associations tested using unconditional logistic regression adjusted for key covariates (age, gender, relationship, rural background, rural training, rural-orientation of the students’ universities, rural club, student scholarships (John Flynn or RAMUS)). | In the multivariate model, independent predictors of rural practice intention at course completion included (OR [95% CI]): intention to work in a rural location at course commencement (6.04 [4.82-7.56]), rural background (2.33 [1.59-3.41]), late rural immersion (1.32 [1.00 – 1.74]) and late remote immersion (1.77 [1.25, 2.49]). | Response rate 73%, n=3268/4486 after missing data. The duration of immersion measured in days in different settings and according to timing, not easily relatable to total duration of immersion. Did not specify how handled international students. No measurement of actual rural practice outcomes. |
| Kitchener et al 2015 (30)  Queensland | Longlook: initial outcomes of a longitudinal integrated rural clinical placement program | 472 medical students in year 3 and 4 of Griffith University and working as Queensland interns 2010-2013 | Longitudinal study using university and health department data, to test the effect of rural immersion on rural internship location using logistic regression analysis (adjusted for rural background, gender and cohort). | In 2013, 67%, n=31/46 rurally-immersed worked in rural Queensland internships compared with 15%, n=63/426 of metropolitan-trained (11.91, [6.08-23.32]). Immersed only in year 3, 81%, n=21/26 were rural interns, compared with 6 of 6 immersed for 18 months (3^rd^ and 4^th^ year) and 57%, n=16/28 immersed only 6 months in 4^th^ year. | Population study of Griffith University graduates in Queensland internships but small number (n=48 students) immersed, subsequently in Queensland internships. Few key covariates adjusted for. Did not specify how handled international students. |
| Kondalsamy-Chennakesavan et al 2015 (29)  Queensland | Determinants of rural practice: positive interaction between rural background and rural undergraduate training | 1572 medical students from University of Queensland who completed course between 2002-2011 | Cross-sectional online or hard copy questionnaires between Dec 2012 and Oct 2013 exploring influence of immersion on 2013 rural practice. Analysed using multiple logistic regression (adjusted for parent's rural background, partnership status, partner's rural background, rural return of service obligation, boarding school attendance and year off after high school). | 19%, n=90/478 metropolitan-trained working rurally, versus 42%, n=115/276 rurally-immersed (P<0.001). 61%, n=73/119 rural- versus 27%, n=42/157 metropolitan background worked rurally. Independent predictors of rural work (multivariate model) were: immersion 1 or 2 years’ (1.84 [1.21–2.82] and 2.71 [1.65–4.45] respectively), rural background (2.30 [1.57–3.36]), partner has rural background (3.08 [1.96–4.84]), being single (1.98 [1.28–3.06]), a bonded scholarship (2.34 [1.37–3.98]). Immersion of metropolitan background students 1 or 2 years not significant, though 2 years’ immersion approached significance (1.83 [0.91-3.67], P=0.09. | Response rate 48%, n=754/1572, self-reported rural practice - not validated. Did not specify how handled international students or range of types of rural return of service obligations like Queensland specific scholarships bonding students to work in rural health services. |
| Lee et al 2011 (20)  Australian Capital Territory | Initial evaluation of rural programs at the Australian National University: understanding the effects of rural programs on intentions for rural and remote medical | 88 year 4 medical students in 2008 who were in the Australian National University’s RCS at some point during the 4 year course, all rurally-immersed | Anonymous and voluntary 33-item online questionnaire about factors affecting student intention for rural work at course completion. Univariate analyses only. | 65%, n=26/40 respondents considered working in a rural/remote area at course commencement and 82%, n=33/40 at course completion. | 45% response (n=40/88). Small, single graduate cohort in study. Limited to interest in rural practice only, no adjustment for key confounders. International students excluded. No metropolitan-trained control group. |
| McDonnel Smedts et al 2007 (21)  South Australia/Northern Territory | Clinical training in the Top End: impact of the Northern Territory (NT) Clinical School, Australia, on the Territory's health workforce | 452 Flinders University medical students who completed 4-year course 1999-2005, and had participated in NT RCS (outer regional location) immersion or not | Using a database about students and their training and internship location, explored how NT rural immersion is associated with NT internship. Univariate t-tests and chi-squared analyses. | 54%, n=52/96 of students who attended the NT clinical school completed internships in the NT compared to 4%, n=13/356 of students not trained at the NT clinical school. Students spending >20 weeks of their final year in NT were nearly 20 times more likely to do NT internship than those who spent <20 weeks. | Unspecified number of students excluded due to incomplete data, so response rate unreported. Limited to exploring internship outcomes, no adjustment for key confounders. Did not specify how handled international students. No metropolitan-trained control group. |
| McDonnel Smedts et al 2008 (35)  South Australia/Northern Territory | Efficiency of clinical training at the Northern Territory Clinical School: placement length and rate of return for internship | 734 Flinders university medical graduates who participated in NT (rural) immersion between 1998 and 2007 | Using university data about weeks spent training in the NT and associations with rural internship in NT, tested using logistic regression analyses (covariates were different training periods and timings of training). | Compared with NT placement length <20 weeks in final year, longer duration >20 weeks final year was a significant predictor of an NT internship (37.14, [4.88–282.97]) as was > 20 weeks in year 3 (16.88, [5.17–55.10]). | 93% of graduates, n=683/734 who had a NT placement were included in analysis. Limited to internship outcomes, no adjustment for key confounders. Did not specify how handled international students. No metropolitan-trained control group. Most exposure was <20 weeks, small sub-groups in other exposures, and wide confidence intervals for estimate odds ratios. |
| Playford et al 2012 (41)  Western Australia | Rural Undergraduate Support and Coordination, Rural Clinical School, and Rural Australian Medical Undergraduate Scholarship: rural undergraduate initiatives and subsequent rural medical workforce | 682 medical students from University of WA enrolled in first year medicine from 1997-2001 of whom 490 were able to be linked with WA internship data | Using university and health service data for 2003-2007 to describe rural internship and PGY 2 outcomes for students who were rurally-immersed for one year or had other rural exposure during medical degree (6 week). Methods of analysis not defined and comparison groups hard to distinguish. Univariate analyses only. | 156/490 (32%) of all graduates working in WA took at least one rural rotation during internship. 45%, n=34/76 of those immersed did rural internship, compared to 29%, n=122/414 not immersed. 82/297 (28%) of tracked PGY2 doctors had at least one rural rotation; 69%, n=22/32 of if had been immersed versus 23%, n=60/265 of those not immersed (3.0 [1.6–5.6]). | Limited to doctors remaining in WA post-medical degree (72%, n=490/682 tracked to PGY 1; 44% 297/682 tracked to PGY 2). No adjustment for key confounders, although rural return of service obligations were not introduced until after the study period. Did not state how treated international students. |
| Playford et al 2014 (26)  Western Australia | Impact of the Rural Clinical School of Western Australia on work location of medical graduates | 1116 medical students who participated in the WA RCS between 2002 and 2009 | Using information about rural immersion and rural work location in 2013 for students with rural or metropolitan background, tested using multiple logistic regression (adjusted for rural background, sex and age). | 16%, n=42/258 immersed were working rurally compared with 5%, n=36/759 controls (P=0.001). 21%, n=13/63 with rural background working rurally vs 15%, n=29/195 metropolitan background. In final logistic regression rural and metropolitan-background immersed students were associated with rural work (7.5, [3.5–15.8] and (5.1 [2.9–9.1] respectively) compared to metropolitan background and metropolitan trained students. | 1017/1116 (91%) were included in the study. Did not adjust for students with rural return of service obligations. Did not state how treated international students. |
| Playford et al 2015 (27)  Western Australia | Longitudinal rural clerkships: increased likelihood of more remote rural medical practice following graduation | 3282 medical students from University of WA who completed course 1980-2011, limited to 324 working rurally (inner regional to very remote) in 2014 | Using university and workforce registration data to explore whether rural immersion increase likelihood of working in outer regional or remote, versus inner regional locations. 3 groups: *historical controls* -200 who entered medicine prior to rural immersion programs; *not-immersed* - 63 metropolitan-trained when immersion available and; *immersed* - 61. Multiple logistic regression analyses (accounting for sex, remoteness of town of rural background). | 46%, n=94/203 of *historical controls* and 52%, n=33/63 of *not-immersed* were currently working in outer regional/very remote locations as opposed to inner regional, compared with 79%, n=48/61 of those *immersed*. Multivariate analyses showed rural immersion predicted a more than 4-fold increase in the odds of practicing in an outer regional or remote compared with an inner regional area (4.11 [2.04-8.30]). | 92%, n=3020/3282 could be followed up but only 324 were working rurally from this group. International students excluded. Not all key covariates were adjusted for. |
| Playford et al 2016 (24)  Western Australia | Creation of a mobile rural workforce following undergraduate longitudinal rural immersion | 417 medical students who participated in University of WA RCS 2004-2013, all rurally-immersed | Annual mailed survey to explore work location at PY 1- 10, in 2013, and cumulative rural work by RCS graduates, excluding locum work of less than one week. Univariate analyses only. | 17%, n=63/ 367 working rural full time in 2013. Another 22%, n=81/367 had worked for some time in rural area in 2013 (56% for 2-12 weeks n=45/81). Remainder 61%, n=223/367 only worked metropolitan in 2013. The average proportion of postgraduate time (2003-2013) graduates had spent working rurally was 21%. | 88%, n=367/417 contacted. No adjustment for key confounders. No metropolitan-trained control group. Did not state how treated international students. |
| Ray et al 2015 (34)  Queensland | James Cook University’s rurally orientated medical school selection process: quality graduates and positive workforce outcomes | 879 James Cook University (JCU) medical students (2000-2008) who participated in 6 years wholly rural medical course | University and workforce data used to explore whether rurality of town of origin associated with rural internship and rural work in PGY1-9. ANOVA and univariate logistic regression analysis, including rural return of service obligations, rural home town, interview score for course entry. | Rural internship was associated with 2.6 times the odds of having either a rural hometown (P<0.001; 95%CI 1.9–3.6) or remote hometown (odds 1.8) (P=0.139, 95%CI 0.9–3.6) compared with a metropolitan/inner regional hometown, while at PGY 9 these odds were 4.2 (P=0.017, 95%CI 1.3–13.8) and 9.5 (P=0.071, 95%CI 0.8–109.2), respectively. | Population study with 97%, n=744/768 JCU graduates included in analysis. No adjustment for key confounders. No metropolitan-trained control group. Did not state how treated international students. |
| Schauer et al 2013 (25)  Queensland | Factors driving James Cook University Bachelor of Medicine, Bachelor of Surgery graduates' choice of internship location and beyond | 261 JCU medical students who participated in 6 years of wholly rural medical course, completed 2005-2009, provided contact details | Cross-sectional online questionnaire about location of internship and current practice location in 2012. Univariate analyses only. | 34%, n=59/175 undertook internships in metropolitan ASGC-RA 1 location and 66%, n=116/175 rural, but in 2012, 46%, n=80/175 working in a metropolitan location, associated with surgical and paediatrics training (P=0.007 and P=0.063 respectively) and 95/175 in rural associated with general practice and rural generalist training (P=0.010 and P=0.001 respectively). | 67% response rate (n=175/261). Reported proportions in metropolitan internships only. Follow up PGY 3-PGY7. No adjustment for key confounders. |
| Sen Gupta et al 2013 (15)  Queensland | James Cook University MBBS graduate intentions and intern destinations: A comparative study with other Queensland and Australian medical schools | 445 JCU medical students (in last year of 6 years of wholly rural medical course) 2005-2010 | Longitudinal study tracking intern locations as combined cohort based on course exit survey. Compared with intern data via a course exit survey of n=1457 graduates from all other medical schools and n=720 graduates of other medical schools in Queensland. Univariate analyses only. | 67%, n=194/292 of graduates undertook internship in a rural area compared with the average of 17%, n=347/2048 from other Australian medical schools (10.0, [7.6-13.1]) and 32%, n=222/696 from other Queensland medical schools (4.4 [3.3-5.8]). 47%, n=136/292 JCU graduates undertook internship in outer regional centres compared with 5%, n=104/2048 from other states’ medical schools (16.6 [12.3-22.5]) and 13%, n=90/696 elsewhere in Queensland (6.0 [4.3-8.2]). | 66% (n=292/445) response rate to the JCU exit survey. The national data were derived by averaging outcomes for 6 other medical schools in other states (not Tasmania), but no information provided about other universities' rural immersion. Outcome suggest state bias as good outcomes for other Queensland medical schools. No adjustment for confounders. No metropolitan-trained control group. |
| Sen Gupta et al 2014 (33)  Queensland | Positive impacts on rural and regional workforce from the first seven cohorts of James Cook University medical graduates | 536 medical students from JCU (wholly rural training) completing course in 2005-2011 | Longitudinal tracking graduates practice locations from 2006-2012, measured as graduate-years practising and exploring effects by rural and metropolitan background of students using univariate analyses only. | 69%, n=284/413 with rural background did internship in rural location versus 43%, n=35/82 metropolitan-background graduates. Of whole cohort, 20%, n=108/536 did internship in least rural (ASGC-RA2) locations and 43%, n=229/536 in most rural (ASGC-RA 3-5). Of all of the graduate’s postgraduate years (1-7), 40%, n=778/1925 were in cities, 18%, n=353/1925 in inner regional and 40%, n=734/1925 in outer regional compared with other Australian medical graduates (77%, n=2331/3034 major cities, 307/3034 inner regional and 6%, n=174/3034 outer regional). Rural background graduates spent proportionally less years (PGY 1-7) in cities (31%-39%) than those with metropolitan-background (57%-66%). | Data on practice location 85% complete, n=456/536. No adjustment for key confounders (yet large proportion rural background students in the cohort). Did not state how treated international students. |
| Shires et al 2015 (28)  Tasmania | Regional universities and rural clinical schools contribute to rural medical workforce, a cohort study of 2002 to 2013 graduates | 974 medical students completing course at University of Tasmania 2002-2013 | Longitudinal study tracking graduate training location and 2014 work location based on national workforce registration data, comparing those who were immersed in the RCS (a town 25,000 population one year) with those wholly trained in large regional centres. Univariate analyses only. | Larger proportion of immersed students working in an AGSC-RA 2–5 area compared with those trained in large regional centres (57%, n=106/185 vs 49%, n=333/684, χ2(1)=4.3, P=0.038; 1.4 [1.0–2.0]). This proportion increased for more remote locations (RA3-5) (28%, n=51/185 vs 7%, n=49/684, χ2 (1) =59.5, P<0.0001; 4.9 [3.2–7.6]). | 89%, n=869/974 graduates could be tracked to a work location in 2014. 328 were working in Tasmania and 541 in another state. No adjustment for key confounders. Did not state how treated international students. |
| Wilkinson et al 2004 (22)  Queensland | Preliminary evidence from Queensland that rural clinical schools have a positive impact on rural intern choices | Origin of hospital interns in Rockhampton, Toowoomba and Mackay hospitals, Queensland, 2002-2005 | Time series analysis of numbers of interns based on where students trained (Rockhampton, Toowoomba are RCS sites) versus MacKay (not an RCS site). Not statistically tested. | Limited specific quantitative data exploring trends. Raw numbers suggest immersed students in Rockhampton and Toowoomba take up internships there. But immersed students also work in Mackay. | Small scale study, no statistical analysis. Suggestive only of intern patterns in three hospitals. Limited to internship. |
| Woolley et al 2016 (37)  Queensland | James Cook University’s decentralised medical training model: an important part of the rural workforce pipeline in northern Australia | 768 JCU medical students completing course (2005-2012) who had done wholly rural medical course (up to PGY9) | Association between intern location (2006-2013) and current location in 2014 and the RCS they trained in (Townsville, Cairns, Mackay and Darwin). Multiple logistic regression accounted for town of origin Queensland, health rural scholarship (tied to 6 years employment in rural health service at graduation), other rural return of service obligations. | The RCS attended associated with internship location (ORs for all sites 7.1-85.7) and current practice location (for three sites ORs 2.9-3.8, excluding Darwin). Both internship and current location associated with student town of origin. Multivariate model no association with bonded rural return of service obligations for both internship and current practice location. | Practice location identified for 84%, n=642/768 graduates. Did not account for all potential confounders but specifically explored rural background by proximity to work location. Confidence intervals for some estimates were very wide, as some subgroups (e.g. Darwin RCS attendance) were small. |
| Worley et al 2008 (36)  South Australia | Vocational career paths of graduate entry medical students at Flinders University: a comparison of rural, remote and tertiary tracks | 150 3^rd^ year medical students from Flinders (1998-2000), undertaking the parallel rural community curriculum or one year of a regional tertiary referral hospital or neither | Hard copy survey to explore effects of different immersion settings on rural career orientation (training for rural work or working in rural area) by graduates in 2005, explored using multiple logistic regression (adjusted for age and rural background) | Immersion in the parallel rural community curriculum was associated with 19.1 times the odds (95% CI 3.4-106.3, P<0.001) of a rural career compared with graduates who were wholly trained in metropolitan areas. If trained in regional hospital, 4.3 times the odds (95% CI 1.2-14.8, P=0.026) of rural career. | 74/150 students responded (49%): Small sub-groups: 16/30 respondents from regional hospital immersion and 13/15 community immersion. Wide confidence intervals. Rural work outcome poorly defined encompassing "in, or training for rural practice”. Did not state how treated international students. Not adjusted for rural return of service obligations. |

^a^ Papers arranged in alphabetical order according to first author surname. RCS – Rural Clinical School where rural immersion takes place; Interns – first postgraduate year of medicine; RRMA Rural and Remote Metropolitan Areas; RAMUS Rural Australian Medical Undergraduate Scholarship; PGY Postgraduate Year following completion of the medical course; ASGC-RA Australian Standard Geographical Classification – Remoteness Areas

^b^ OR odds ratio; CI confidence interval - (OR [95% CI]); χ2 chi-squared test

^c^ No studies provided justification for the time-point at which rural practice was measured
